# Role of miRNAs in Alzheimer’s Disease and Possible Fields of Application

**DOI:** 10.3390/ijms20163979

**Published:** 2019-08-15

**Authors:** Serena Silvestro, Placido Bramanti, Emanuela Mazzon

**Affiliations:** IRCCS Centro Neurolesi “Bonino-Pulejo”, Via Provinciale Palermo, Contrada Casazza, 98124 Messina, Italy

**Keywords:** Alzheimer’s disease, miRNA, in vivo experimental models

## Abstract

miRNAs (or microRNAs) are a class of single-stranded RNA molecules, responsible for post-transcriptional gene silencing through binding to the coding region as well as 3′ and 5′ untranslated region of target genes. About 70% of experimentally detectable miRNAs are expressed in the brain and some studies suggest that miRNAs are intimately involved in synaptic function and in specific signals during memory formation. More and more evidence demonstrates the possible involvement of miRNAs in Alzheimer’s disease (AD). AD is the most common form of senile dementia, a disease that affects memory and cognitive functions. It is a neurodegenerative disorder characterized by loss of synapses, extracellular amyloid plaques composed of the amyloid-β peptide (Aβ), and intracellular aggregates of hyperphosphorylated TAU protein. This review aims to provide an overview of the in vivo studies of the last 5 years in the literature describing the role of the different miRNAs involved in AD. miRNAs hold huge potential as diagnostic and prognostic biomarkers and, at the same time, their modulation could be a potential therapeutic strategy against AD.

## 1. Introduction

Alzheimer’s disease (AD) is a progressive neurodegenerative disease and it is the most common cause of dementia worldwide. It is characterized by neuronal death, loss of synaptic function, and atrophy in different brain areas, with consequent loss of cognitive functions and memory [1,2,3]. The AD is characterized by neuritic (or amyloid) plaques and neurofibrillary tangles (NFTs) [1]. The diagnosis of AD is performed through neurological investigations, the administration of cognitive tests, the performance of specific neuroimaging tests, and cerebrospinal fluid tests [4]. Neuritic plaques are extracellular accumulations of beta-amyloid (Aβ). Many studies have suggested that Aβ regulates neuronal and synaptic activities. Its accumulation in the brain plays a crucial role in initiating the disease and triggering a complex pathological cascade, which leads to neuronal damage [5]. Aβ peptide derives from the enzymatic proteolysis of the amyloid precursor protein (APP), a protein that physiologically plays an important role in brain homeostasis [6]. A first pathway involved in APP processing is the non-amyloidogenic α-secretase-mediated pathway. APP cleavage by α-secretase generates sAPPα, a soluble molecule that has a probable neuroprotective function. Indeed, this peptide plays an important role in the plasticity and survival of neurons and in the protection against cytotoxicity [7,8]. In healthy subjects, the degradation process of the APP appears to be operated mainly by α-secretase. In contrast, in the subjects with AD, the Aβ peptide is produced following a β- and γ-secretase-mediated amyloidogenic pathway [9]. In this way the APP is mainly processed by the β-secretase cleaving enzyme 1 (BACE1) which operates the first cut, generating sAPP-β and a fragment consisting of 99 amino acids. The γ-secretase further cleaves this fragment from which a peptide of 40 amino acids and one peptide of 42 are generated, called Aβ_1–40_ and Aβ_1–42_, respectively [10,11]. Aβ_1–42_ is more hydrophobic, amyloidogenic, and toxic [12,13]. In the extracellular space, Aβ tends to deposit in insoluble fibrillar aggregates, which lead to the formation of the so-called amyloid plaques. These neuronal plaques trigger a reactive inflammatory process that irreversibly damages neurons [14,15]. Another protein involved in AD is the TAU, which under conditions of hyperphosphorylation leads to the formation of NFTs in the cell body of neurons. TAU is a protein that stabilizes microtubules and promotes vesicular-mediated transport. In neurons, microtubules are essential for maintaining neuronal structure, axonal transport, and neuronal plasticity [16]. In physiological conditions, the functioning of the TAU is given by a perfect balance between phosphorylation and dephosphorylation. In the etiopathogenesis of AD, however, excessively phosphorylated TAU causes the formation of NTFs with consequent destabilization and neuronal death [17]. Two forms of AD have been identified: Familial AD (FAD), also known as AD early-onset and sporadic AD (SAD), characterized by late-onset. About 70% of the risk of developing FAD may be due to mutations in the *APP*, *PSEN1*, and *PSEN2* genes [18]. The *APP* gene encodes for the amyloid precursor protein (APP), while the *PSEN1* and *PSN2* genes encode for presenilin 1 (PSEN1) and presenilin 2 (PSN2) proteins [19]. PSEN1 and PSEN2 are enzymes belonging to the class of proteases A that regulate the functions of the enzyme γ-secretase, responsible for cutting the amyloid protein. Mutations in *PSEN1* and *PSEN2* genes lead to the accumulation of Aβ. Instead, late-onset AD is mainly associated with a polymorphism in the *APOE* gene encoding for apolipoprotein E (APOE), a protein involved in lipid metabolism [20,21]. In particular, the APOE4 isoform can influence the pathogenesis of AD by promoting the conversion of Aβ into a fibrillar form and its deposition. In addition, APOE4 also appears to have adverse effects on Aβ clearance. Aβ is normally transported through the blood–brain barrier via protein 1 associated with the LDL receptor (LRP1) in the blood for the possible degradation in the liver. The APOE4 isoform has a low specificity toward LRP1, consequently, it slows the clearance of Aβ and favors its accumulation [22]. Furthermore, recent studies have shown that some modifying factors such as lifestyle and diet reduce the risk of occurrence of AD. Specifically, physical activity stimulates neurogenesis, synaptic plasticity, and the production of neurotrophins [23]. In addition, both physical activity and a diet enriched with polyphenols, coumarin, catechins, resveratrol, vitamin E, and omega-3 fatty acids have an anti-inflammatory effect and reduce oxidative stress, two important factors of the etiopathogenesis of AD [24,25]. MicroRNAs (miRNAs), a large family of small noncoding RNAs, are important regulators of gene expression at the transcriptional and post-transcriptional level. They can regulate gene silencing. About 70% of all identified miRNAs are expressed in the brain [26]. Several studies, in fact, show that the misregulation and alterations of specific miRNAs could contribute to the etiopathogenesis of AD [27,28]. The discovery that miRNAs interfere with the expression of genes involved in AD has suggested the development of strategies that provide for the use of miRNAs as therapeutic agents. Specifically, the researchers have used an approach that involves the use of single-stranded antisense oligonucleotides, which block the transcription of miRNAs involved in AD. Alternatively, double-stranded synthetic oligonucleotide miRNAs can be used. In this way, it enhanced the expression of some miRNAs that counteract Aβ and TAU accumulation [29]. One of the major challenges in the field of miRNA therapy in AD treatment remains effective delivery to target the brain. In particular, the delivery of miRNAs into the central nervous system is very critical as the biochemical and biological properties of the blood-brain barrier. In the preclinical studies delivery of miRNAs into the brain is performed frequently through intracerebroventricular injections by the use of an implanted catheter connected to a pump [30]. Alternatively, the intrathecal route can be used, delivering miRNAs in the subarachnoid space. This review aims to provide an overview of the in vivo studies that describe the role of different miRNAs in AD and their possible fields of application in the last 5 years.

## 2. miRNA

MiRNAs (or microRNAs) are small noncoding RNAs (21–25 nucleotides) that play a significant role in the post-transcriptional regulation of gene expression in eukaryotes [31]. The miRNAs exert their action in post-transcriptional gene silencing, binding to the coding region as well as 3′ and 5′ untranslated region (UTR) of the messenger RNAs (mRNAs). miRNAs perform a “hetero-silencing” mechanism, blocking the translation or degrading target mRNA. The same miRNA can target different mRNAs; at the same time, a single mRNA can be regulated by different miRNAs. The transcription of the primary miRNAs (pri-miRNA) is carried out by the RNA polymerase II, toward which they show a particular affinity. The affinity for RNA polymerase II must be attributed to the presence in the primary transcript of promoters that contain typical characteristics of RNA polymerase II [32]. The miRNAs originate from a long transcript characterized by a double-stranded structure known as pri-miRNA. In the nucleus, the pri-miRNA is recognized by the Pasha protein (Partner of Drosha) which is associated with RNAase type III Drosha. Pasha orients the catalytic domain of Drosha so as to allow the enzyme to cut the pri-miRNA, obtaining transcripts of about 70 nucleotides called miRNA precursors (pre-miRNA) [33]. Subsequently, the pre-miRNAs complexed by RAN-GTP proteins and through the exportin 5 are transported from the nucleus to the cytoplasm [34,35]. In the cytoplasm, the pre-miRNA undergoes a second cut by another RNase III, Dicer, generating RNA molecules of about 22 nucleotides, called miRNAs. After cleavage, a single strand of miRNA is incorporated into the complex known as “RNA-induced silencing complex” (RISC), while the other strand is degraded. Within RISC, mature miRNA binds to and regulates specific target mRNAs [36]. miRNAs can induce post-transcriptional gene silencing using two different mechanisms depending on the complementarity between the miRNA and its mRNA target. The miRNAs that bind perfectly complementary areas of their target mRNA induce its degradation by deadenylation, cap removal, and exonucleolytic digestion of mRNA. In contrast, miRNAs that bind mRNA with imperfect complementarity cause a translation block. This block can occur by repression of the translation during the initial phase or during the elongation phase. Alternatively, miRNAs can repress translation even by inducing premature ribosome detachment [37].

miRNAs are involved in the development of a variety of pathological conditions such as cardiovascular diseases, cancer, arthritis, cataracts, osteoporosis, diabetes/obesity, and hypertension. They are also implicated in various neurodegenerative diseases such as AD, Huntington’s disease, Parkinson’s disease, amyotrophic lateral sclerosis, and schizophrenia [38].

The fine regulation of genes associated with pathologies through miRNAs could be an important mechanism to maintain neuronal homeostasis and allow the neuronal circuits to respond adequately to environmental insults [39].

## 3. miRNA and Experimental Models of Alzheimer’s Disease

Several studies evaluated the role of miRNAs in AD mouse models. The most used AD animal models are the APP/PSEN1 transgenic models, developed after the discovery that mutations of the *APP* and *PSEN1* genes are responsible of the familial forms of AD.

Lin, et al. [40] investigated the effects of osthole on miRNA expression and the consequent influence in AD. Osthole, a coumarin derivative, has several anti-inflammatory, anti-apoptosis, anti-oxidative properties [41] and it is already known to have a therapeutic role in AD [42]. The effects of osthole were evaluated in APP/PSEN1 mice. APP/PSEN1 are double transgenic mice expressing a chimeric precursor protein of human amyloid (Mo/HuAPP695swe) and a mutant human presenilin 1 (PS1-dE9). The results of the in vivo study showed that the osthole induced the upregulation of miRNA-101a-3p. Furthermore, western blot analysis performed in hippocampal and cortex extracts of mice revealed that the expression of APP protein and Aβ decreased in the experimental group treated with osthole. At the same time, an in vitro study was performed using human neuroblastoma cells SH-SY5Y transfected with GFP-APP595/596 lentivirus with overexpression of APP. According to the results obtained from the in vivo study, treatment with osthole showed an increase in the expression of miRNA-101a-3p, a reduce cell damage, and an increase in viability. In order to demonstrate the mechanism by which osthole influences miRNA-101a-3p by regulating the expression of the APP protein, the same cells were transfected with the miRNA-101a-3p inhibitor. In this experimental condition, it was observed an increase in the expression of the APP and Aβ protein.

Higaki et al. [43] conducted a study in order to correlate the differential expression of the miRNA-200 family (miRNA-200a, -141, -429, -200b, -200c) in the initial phases of AD in the mouse brain Tg2576. Tg2576 mice overexpress the APP protein (Swedish *KM670/671NL* mutation). Analysis of the total RNA microarray extracted from cortical tissues of mice revealed that miRNA-200a, -141, -429, -200b, and -200c were upregulated only in Tg2576 mice of 10 months of age. These results suggest that some miRNAs may respond to the early Aβ accumulation. In addition, an in vitro study was conducted on primary murine neuronal cells (PMNC) isolated from the cortical tissues of mice in order to verify if the expression of miRNA-200b and miRNA-200c are altered in response to neuronal damage induced by Aβ_1–42_. The treatment with Aβ of the PMNC cells induced the upregulation of miRNA-200b or -200c. Subsequently, the cells were transfected with miRNA-200b/c demonstrating that the upregulation of miRNA-200b and miRNA-200c reduced the secretion of Aβ in conditioned medium. In order to evaluate the effect of miRNA-200b/c in vivo, Tg2576 mice were treated with miRNA-200b/c by intracerebroventricular injection. This experiment confirmed what was obtained in vitro, suggesting that miRNA-200b and miRNA-200c may be potential therapeutic targets in AD.

Liu et al. [44] conducted a study to evaluate the expression of miRNA-220b, miRNA-135a, and miRNA-429 in the hippocampus of APP/PSEN1 transgenic mice. Microarray miRNA analysis showed that these miRNAs were significantly upregulated. In addition, this analysis was supported by bioinformatics tools that disclose the potential interaction between APP and BACE-1, an enzyme responsible for the production of Aβ. In order to verify the effects of these miRNAs on the expression of APP and BACE-1, an in vitro study was also conducted using primary hippocampal neurons isolated from mice and SH-SY5Y cells transfected with oligonucleotides miRNA-135a, -200b, and -429. The results obtained show that miRNA-135a repressed the expression of BACE-1, whereas miRNA-200b and miRNA-429 decreased the expression of the APP. Instead, cells transfected with the miRNA-135a inhibitor oligonucleotide induced a significant upregulation of BACE-1. Whereas, in cells transfected with miRNA-200b and miRNA-429 inhibitor oligonucleotides, a significant upregulation of the APP protein was observed. These data were supported by the clinical study that showed a decrease only of miRNA-135a and -200b levels in serum and cerebrospinal fluid of AD patients compared to healthy subjects.

Zhang et al. [45] investigated the involvement of miRNA-200a-3p upregulation in Aβ-induced neuronal apoptosis by inhibiting the silent information regulator transcript-1 (SIRT1) in APP/PSEN1 mice. SIRT1 can deacetylate histone, nonhistone proteins, other transcription factors and is involved in the regulation of cellular senescence, gene transcription, energy balance, and oxidative stress. The effects of SIRT1 could act upstream and downstream of Aβ [46]. An increase in SIRT1 is typically related to the neuroprotection in neurodegenerative disorders [47]. In hippocampus of APP/PSEN 1 mice and in vitro in pheochromocytoma PC12 cells treated with Aβ_25–35_, elevated expression of miRNA-200a-3p and a reduced level of SIRT1 were found. In particular, PC12 cells treated with Aβ_25–35_ showed a higher level of expression of cleaved-caspase-3, while these levels decreased when the cells were transfected with a miRNA-200a-3p inhibitor. In addition, when instead PC12 cells were transfected with plasmid vectors that host the SIRT1 and subsequently co-transfected with miRNA-200a-3p inhibitors, it was observed that the suppression of miRNA-200a-3p attenuates Ab_25–35_-induced apoptosis.

miRNA-29a, miRNA-29b, and miRNA-29c are a class of miRNAs known to regulate BACE-1 expression and pathogenesis of AD [48]. Yang et al. [49] studied the role of miRNA-29c in AD. Peripheral blood samples from AD patients showed a decrease in miRNA-29c expression levels and a significant increase in BACE-1 expression compared to healthy subjects. In addition, the researchers evaluated the role of miRNA-29c in in vitro and in vivo studies. Primary hippocampal neurons were obtained from senescence-accelerated mouse-resistant 1 (SAMR1) and were transfected with miRNA-29c or miRNA-29c inhibitors. Results showed that miRNA-29c overexpression significantly reduced BACE-1 expression levels. However, an inverse result was obtained when the cells were treated with the miRNA-29c inhibitor. This molecular mechanism was subsequently confirmed in in vivo study, using AD senescence-accelerated mouse prone 8 (SAMP8). SAMP8 mouse is a mutant of spontaneous crosses, characterized by an accelerated aging phenotype. After mimic injections of miRNA-29c in the hippocampus of mice showed significantly increased levels of miRNA-29c compared to control mice. The in vivo study confirmed the in vitro results showing that miRNA-29c upregulation significantly reduced the expression levels of BACE-1 and Aβ proteins.

Furthermore, Zong et al. [50] studied mRNA-29c levels in the APP/PSEN1 mouse model. The results showed that miRNA-29c levels were significantly increased in the hippocampus of mice. However, they were significantly decreased in the frontal cortex. This discrepancy in the miRNA-29c level between the hippocampus and the frontal cortex may indicate that the various miRNAs are expressed in a spatially controlled manner. The immunohistochemical observation also revealed that in the hippocampus of the young APP/PSEN1 mice, where the levels of miRNA-29c were high, neuron navigator 3 (NAV3) protein levels were decreased. NAV3 is expressed predominantly in the central and peripheral nervous system and appears elevated in the AD brain. Although the biological function of the mammalian NAV3 protein in the brain remains totally unknown, it appears to play a key role in axon guidance [51]. The inverse expression of miRNA-29c and NAV3 was also observed in neuroblastoma (Neuro2A) cells that were transfected with the miR-29c and successively with the probe directed against miRNA-29c. It is not yet clear whether the enhanced expression of NAV3 in the AD brain reflects some pathogenetic changes or is attributable to a defense mechanism against neurodegenerative events. However, the results of the present study may show that miR-29a underexpression affects neurodegenerative processes by increasing the regulation of NAV3 and other miR-29a targets, such as BACE-1.

The study conducted by Jiang et al. [52] aimed to analyze the effect of miRNA-137 in APP/PSEN1 transgenic mice. MiRNA-137 is a regulator of neuronal development and cognitive function, which also appears to be involved in AD [53,54]. The researchers also evaluated the regulatory effect of miRNA-137 on the transcription of its potential target gene, calcium voltage-gated channel subunit alpha-1 C (*CACNA1C*). *CACNA1C* is a gene that encodes the alpha 1C subunit of the voltage-dependent calcium channel of type L CaV1.2 [55] and it is responsible for the regulation of intracytoplasmic calcium (Ca^2+^) in neurons. As has been shown, an increase in the Ca^2+^ influx through CaV1.2 contributes to neuronal dysfunctions, as found in AD [56]. The results revealed a decrease in miRNA-137 levels and an increase in Aβ and CACNA1C protein levels in the hippocampus and in the cerebral cortex of the AD mouse. The role of miRNA-137 in *CACNA1C* expression was also validated by evaluating the expression of *CACNA1C* in human SH-SY5Y neuroblastoma cells treated with or without Aβ_1–42_ and subsequently transfected with miRNA-137 mimics or miRNA-137 inhibitors. The results confirmed the data obtained in the in vivo study demonstrating that the downregulation of miRNA-137 determines an increase in Ca^2+^ levels and a reduction of Aβ_1–40_ and Aβ_1–42_. These results indicate that an increase in this miRNA could cause a decrease in Ca^2+^ levels in neurons, improving neuronal dysfunctions typical of AD.

The aim of the study conducted by Sierksma et al. [57] was to understand if miRNAs altered are associated with progressive memory deterioration in AD. Transgenic mice for APP (APPtg) and for TAU (TAUtg) were used. MiRNAseq revealed that miR-10a-5p, miR-142a-5p, miR-146a-5p, miR-155-5p, miR-211-5p, and miR-455-5p are commonly upregulated in both APPtg and TAUtg mice. Four of these, miR-142a-5p, miR-146a-5p, miR-155-5p, and miR-455-5p, were upregulated also in patients with AD. Differential analysis of miRNA expression between APPtg and TAUtg mice showed that the overexpression of these miRNAs in TAUtg mice appears starting from four months of age, while the miRNA disorders in APPtg mice mainly occur when Aβ has accumulated at ten months of age. In order to evaluate whether the upregulation of these miRNAs is sufficient to induce the cognitive alterations observed in the APPtg and TAUtg mice, intracerebroventricular injections of a single miRNA oligonucleotide or a mix of the six miRNAs were performed in C57BL/6J. These injections lead to a significant increase in hippocampal expression of each miRNA; however, they do not cause serious cognitive deficits. Therefore, these findings suggest that miRNAs could have a protective effect on AD progression.

The study conducted by Zhou et al. [58] proposed to explore the effect of the interaction between miRNA-330 and guanine nucleotide exchange factor 1 (VAV1), enzymes that catalyze the exchange of guanosine diphosphate (GDP) by guanosine triphosphate (GTP) in Rho GTPases proteins, whose activity is involved in AD. Rho GTPases are involved in synaptic plasticity and showed a relationship with APP synthesis and Aβ production [59]. MiRNA-330 overexpression is associated with brain development and it functions as a regulator of protein expression and regulates the development and progression of the disease [60]. For the study, C57BL/6J mice were used and subjected to intraperitoneal injection with D-galactose for 4 weeks for inducing the AD model. The results obtained showed downregulation of miRNA-330, a significant increase in the expression of the VAV1 and Aβ proteins in the cerebral cortex of the AD mouse. In parallel, an in vitro study was conducted on cells of primary neurons collected from mice. In order to induce overexpression of miRNA-330, primary neurons were transfected with miRNA-330. The first result shows that miRNA-330 overexpression inhibited VAV1, that in turn blocks mitogen-activated protein kinase (MAPK) signaling pathway. MAPK are serine-threonine kinases that mediate a variety of cellular activities including proliferation, differentiation, survival, death, and cell transformation. Activated MAPK signaling pathways contribute to the pathogenesis of AD through various mechanisms including phosphorylation and stabilization of APP, the transcriptional and enzymatic activation of β- and γ-secretase, and the induction of neuronal apoptosis [61]. The second result of the study shows that miRNA-330 upregulation inhibits the production of Aβ always through the MAPK signaling pathway. It appears that MAPK blocks the α7 nicotinic acetylcholine receptor resulting in a reduction in the uptake and accumulation of Aβ [62]. In addition, the upregulation of miRNA-330 suppresses oxidative stress and improves mitochondrial dysfunction always via the MAPK signaling pathway. In this way, upregulation of miRNA-330 inhibits VAV1 and block MAPK signaling pathway determining a reduction of Aβ, a suppress of oxidative stress, and improvement of mitochondrial dysfunction.

miRNA-34a is involved in several AD pathways such as Aβ deposition and cognitive dysfunction. Jian et al. [63] evaluated the role of this miRNA in Aβ production and clearance. For the study, APP/PSEN1 mice were compared to the miRNA-34a knockout APP/PSEN1 (miRNA-34a KO/APP/PSEN1) mice. In APP/PSEN1 mice the level of miRNA-34a increased, consistent with the increase of Aβ. In contrast, in miRNA-34a KO/APP/PSEN1 mice the data showed that the number and area of amyloid plaques were significantly decreased, and a significant reduction in behavioral dysfunction was also observed. This suggests that miRNA-34a upregulation may contribute to the promotion of AD pathology. Furthermore, the role of miRNA-34a in the processing of APP was evaluated. The results showed that the improvement observed in miRNA-34a KO/APP/PSEN1 mice is due to the decrease in APP amyloidogenic processing by β-secretase activity and BACE-1 expression inhibition. In agreement, these results are in line with those of Xu et al. [64] that had observed an increase in miRNA-34a expression in APP/PSEN1 mice, with Aβ production, amyloid plaque deposition, and cognitive deficits. In contrast, the lack of miRNA-34a in knockout mice significantly reduced cognitive deficits and inhibited the amyloidogenic processing of the APP protein. Moreover, researchers also evaluated the expression profile of miRNA-34a-3p and miRNA-34a-5p in the initial phase of AD pathology in APP/PSEN1 mice compared to miRNA-34a KO/APP/PSEN1. Bioinformatics tools predicted that miRNA-34a-5p mainly targeted to *N*-methyl-d-aspartate (NMDA), while miRNA34-3p to α-amino-3-hydroxy-5-methyl-4-isoxazolapropionic (AMPA). AMPA and NMDA are two dynamic receptors on the synaptic membrane involved in synaptic plasticity and information storage [65]. Therefore, the level of mRNA and proteins of multiple subunits of AMPA receptors and NMDA receptors in the hippocampus were determined. It was observed that several types of subunits of AMPA and NMDA receptors were markedly upregulated in APP/PSEN1 mice with miRNA-34a deficiency. Since, in the mature central nervous system, the composition of the subunit of these receptors influences multiple synaptic functions including stabilization, neurotransmission, and even neuronal activity [66,67], a downregulation can decrease synaptic strength, resulting in cognitive impairment. In addition, it appears that this miRNA also appears to be involved in AD anxiety disorder in AD. Specifically, Zhang et al. [68] observed the correlation of miRNA-34a with metabotropic glutamate receptor 7 (GRM7). GRM7 is an excitatory neurotransmitter related to excitatory synaptic transmission, neuronal development and death, synaptic plasticity, and some neurological diseases such as anxiety and depression [69]. The triple transgenic mouse model of AD (3xTg-AD) was used for the study. 3xTg-AD mice contain three mutations associated with FAD (Swedish app, MAPT P301L, and PSEN1 M146V). Behavioral tests were performed on mice that revealed anxiety-like behaviors. Subsequently, the hippocampus was isolated from mice to evaluate the expression of miRNA-34a and e GRM7. The result of this work showed significant miRNA-34a expression in the hippocampus of 3xTg-AD mice and downregulation of GRM7. The results of this study suggest that the anxious behavior observed in 3xTg-AD mice could be related to the dysregulation of miRNA-34a/GRM7 observed in the hippocampus.

Recently, it was shown that a new pathway for improving endogenous repair can be obtained by overexpression of specific miRNAs [70]. In the study conducted by Ghasemi-Kasman et al. [71], it was studied whether the miRNA-302/367 cluster is capable of inducing the conversion of astrocytes into neurons. The miRNA-302/367 cluster, composed of five members, miRNA-367, miRNA-302d, miRNA-302a, miRNA-302c, and miRNA-302b, is involved in the regulation of cell proliferation, differentiation, and reprogramming [72]. Researchers for the study used the AD mouse model induced by intracerebroventricular injection of streptozotocin (STZ). Lentiviral particles of miRNA-302/367 were injected into the hippocampal dentate gyrus together with the intraperitoneal injection of valproate (VPA). VPA has the ability to regulate the expression of neuronal genes related to neurodegenerative disorders, increases neuronal survival and reduces the vulnerability of neural cells to damage. Furthermore, VPA showed the ability to activate neprilysin, the main β-degrading enzyme of amyloid [73]. The results obtained showed that miRNA-302/367 in combination with VPA improved spatial learning and memory that were compromised by STZ injection. Furthermore, two weeks after the injection of miRNA-302/367 clusters, immunostaining against GFAP as an astrocyte marker confirmed that hippocampal activated astrocytes were reprogrammed to neurons in the animal model AD.

The study conducted by Hong et al. [74] was mainly aimed at identifying the level of miRNA-125b circulating in APP/PSEN1 mice and its correlation with cognitive function. Recently, it was shown that circulating miRNA-125b levels were downregulated in AD and could be potential biomarkers of disease [75]. The results of this study showed the serum miRNA-125b level was significantly downregulated in APP/PSEN1 mice. Furthermore, the analysis of expression of circulating miRNA revealed that miRNA-9 and miRNA-191-5p were also downregulated, on the contrary, miRNA-28-3p was upregulated. Moreover, a positive correlation was demonstrated only between miRNA-125b and the decline of cognitive function. In addition, the level of this miRNAs in APP/PSEN1 transgenic mice treated with epigallocatechin gallate (EGCG), an active component of green tea, was also studied. This compound appears to be effective as a potential therapeutic agent for AD, suppressing cognitive dysfunction, increasing learning ability, and reducing damage induced by oxidative stress [76,77]. In mice treated with EGCG the levels of these miRNAs were reversed compared to untreated mice and only miRNA125b showed a positive correlation with cognitive function. In order to further identify the relationship between miRNA-125b and AD, an in vitro model was also performed, treating SH-SY5Y cells with EGCG. The level of miRNA-125b was significantly increased in accordance with the results obtained in in vivo study.

The purpose of the study conducted by Tang et al. [78] was to examine the role of miRNA-139 in spatial memory, fear conditioning, and recognition memory. For the study, the SAMP8 strain was used as an AD model and SAMR1 as a control strain. Compared to control mice, miRNA-139 expression was significantly higher in the hippocampus of the SAMP8 mice. In order to understand the role that miRNA-139 exerts on learning and memory, injections of miRNA-139, miRNA-139 inhibitor, and control miRNA were performed in the dentate gyrus of SAMP8 and SAMR1 mice. The results of the study show that the downregulation of miRNA-139 expression improved spatial memory, the recognition of new objects, long-term memory, and contextual fear conditioning. The study also sought to understand the potential targets of miRNA-139, demonstrating that miRNA-139 inhibits cannabinoid receptor type 2 (CB2) expression. CB2, a membrane marker of activated microglia cells, is involved in synaptic plasticity and neuroprotection and appears to modulate hippocampal function in the context of AD [79]. Furthermore, it was observed that CB2 deficiency affects the ability to activate microglia and the recruitment of macrophages in the brains of AD mice by reducing neuroinflammation [80,81]. In this way, miRNA-139 downregulation by inhibiting CB2 decreases responses to pro-inflammatory stimuli and acts as a regulatory factor in the pathogenesis of AD.

MiRNAs are key factors in development, neurogenesis, and synaptic functions in the central nervous system. The study conducted by Lee et al. [82] investigated the role of miRNA-188-5p in restoration of the synaptic and cognitive deficits in AD. For the study, 5xFAD transgenic AD mice were used. These mice express human *APP* and *PSEN1* transgenes with a total of five AD-linked mutations: three familial mutations of *APP 695* (Swedish, Florida, and London mutation) and two *PSEN1* mutations (M146L and L286V). The results of the study show that the downregulation of miRNA-188-5p expression contributes to the pathogenesis of AD by inducing synaptic dysfunction and cognitive deficits. The expression of miRNA-188-5p was also downregulated in brain tissues from patients with AD compared to healthy subjects of corresponding age. In parallel, an in vitro study was conducted on primary cells of hippocampal neurons treated with Aβ_1–42_ and transfected with miRNA-188-5p oligonucleotides. Treatment with oligomeric Aβ_1–42_ decreased miRNA-188-5p expression in primary cultures of hippocampal neurons. In contrast, it was observed that miRNA-188-5p overexpression alleviated the decrease in dendritic spine density in primary hippocampal neurons exposed to Aβ. This result showed that miRNA-188-5p improved Aβ_1–42_-mediated synaptic dysfunction. In order to validate also in vivo, these results were performed injection of miR-188-5p, subcloned in a lentiviral vector, in the hippocampus of 5XFAD mice. After 3 weeks of injection, the overexpression of miRNA-188-5p has determined the improvement of alterations in cognitive function and synaptic transmission, confirming the results obtained from the in vitro study.

The miRNAs, in addition to influencing the production process of Aβ, are also involved in the phosphorylation and dephosphorylation of the TAU protein, responsible for the formation of NFT. MiRNA-132 is downregulated in the brain of AD patients [83,84]. The aim of the study conducted by Salta, et al. [85] was to characterize the functional impact of the downregulation of miRNA-132 on the AD pathology by identifying the molecular networks underlying these effects. For the experiment, the APP/PSEN1 mice were treated with intracerebroventricular injections of miRNA-132 and miRNA-132 inhibitor. Following these injections, it was observed that the downregulation of miRNA-132 was correlated with an increase of the peptide Aβ and phosphorylated TAU in the hippocampus of APP/PSEN1 mice. In contrast, the upregulation of miRNA-132 caused a reduction in Aβ and phosphorylated TAU. The transcriptome microarray analysis using the prefrontal cortex of patients with AD and healthy individuals demonstrated that inositol 1,4,5-trisphosphate 3-kinase B (ITPKB) was a possible miRNA-132 target. ITPKB is a kinase, known to phosphorylate ERK1/2 leading to an increase in TAU phosphorylation [86,87]. At the same time, ITPKB also influences the activity of BACE-1 which leads to the generation of Aβ. The interaction between miRNA-132/ITKB would explain the effect on reducing Aβ and TAU in APP/PSEN1 mice. In order to confirm the link between miRNA-132 and ITPKB, human embryonic kidney (HEK293) cells overexpressing human APPS were transfected with a miRNA-132 inhibitor oligonucleotide and co-transfected with an ITPKB siRNA oligonucleotide. In vivo experiments confirmed a significant increase in Aβ_1–40_ and Aβ_1–42_. While, the inhibition of ITPKB has determinate a downregulation of ITPKB with a consequent reduction in Aβ, demonstrating the involvement of this kinase in the production of Aβ. This data was further confirmed in in vivo study by intracerebroventricular injections of ITPKB siRNA in mice.

Moreover, in another study conducted by Smith et al. [88], the involvement of miRNA-132 on TAU expression was observed. In particular, the role of the miRNA-132/212 cluster in AD was evaluated in this study. This cluster appears to be downregulated in AD. Numerous studies show that miRNA-132 and miRNA-212 play an important role in synaptic plasticity and memory formation [89]. In in vivo study, miRNA-132/212 knockout mice were used and it was observed that the deletion of miRNA-132/212 caused increased phosphorylation and accumulation of TAU. Autophagy was observed to be one of many factors involved in TAU aggregation [90]. The result of the western blot analysis performed in the brains of these miRNA-132/212 deficient mice confirms that the autophagic process is compromised in the absence of miRNA-132/212. The role of miRNA-132/212 in the regulation of TAU in AD has been further confirmed by treating 3xTg-AD mice with miRNA-132 mimic. After three weeks, the treatment produced better long-term memory deficits and a significant reduction in phosphorylated TAU.

The same research group performed a direct follow-up of the previous study. In this work, Hernandez-Rapp et al. [91] investigated the impact of miRNA-132/212 loss in production, cleavage, and clearance of Aβ. The results showed that the genetic deletion of miRNA-132/212 promotes the production of Aβ and the formation of amyloid plaques in 3xTg-AD-miRNA-132/212 knockout mice. In order to determine the target genes of the miRNA-132/212 cluster with documented roles in the regulation of Aβ metabolism, RNA extracts of hippocampi of 3xTg-AD-miRNA-132/212 knockout mice used for performing RNA sequencing. The results showed that SIRT1, MAPK1/ERK2, and TAU were interesting miRNA-132 targets that were found to be upregulated in 3xTg-AD mice. These results were confirmed in in vitro study using mouse neuro2a-cells and human HEK293 transfected with miRNA-132. Both cell lines were overexpressing APP. The results showed that in the cells transfected with miRNA-132 the Aβ_1–40_ and Aβ_1–42_ levels were reduced, while SIRT1 was downregulated. Normally, an increase of SIRT1 is responsible for neuroprotective effects, therefore, its downregulation determined by upregulation of miRNA-132 contributes to the increase of Aβ and to the deposition of senile plaque in AD.

Wang et al. [92] investigated the relationship between miRNA-146a, TAU-hyperphosphorylation, and rho-associated, coiled-coil-containing protein kinase 1 (ROCK1). The ROCK1 protein appears to be responsible for binding to protein phosphatase and tensin homolog (PTEN) involved in TAU dephosphorylation [93]. SH-SY5Y cells were transfected with miRNA146a. The miRNA-146a overexpression in SH-SY5Y cells caused a significant increase in TAU phosphorylation and simultaneously inhibited the translation of the ROCK1 protein. In addition, in order to evaluate the involvement of PTEN in this process, the SH-SY5Y cells were transfected with siRNA ROCK1. Transfection with ROCK1 siRNA resulted in a decrease in ROCK1 protein levels, reduced PTEN phosphorylation, and increased TAU phosphorylation. These data demonstrate how the expression of miRNA-146a by suppressing ROCK1 caused a reduction in PTEN phosphorylation and TAU hyperphosphorylation. The in vivo study was conducted using 5xFAD mice treated with injections of a miRNA-146a inhibitor in the intra-hippocampal region. This treatment induces an increase of ROCK1 protein level and suppression of TAU hyperphosphorylation. This result demonstrates that miRNA-146a contributes to the pathogenesis of AD and its inhibition could be a valid application as in vivo therapy.

Many miRNAs are implicated in amyloid-β and TAU alterations; however, alterations in synaptic plasticity and neuronal loss are two important features shown in patients with AD. The study conducted by Rodriguez-Ortiz et al. [94] aimed to identify miRNAs that play a critical role in synaptic plasticity underlying the progression of AD. A significant upregulation of miRNA-181 was observed in the dorsal and ventral hippocampus of 3xTg-AD mice with plaques and tangles. Bioinformatics tools were used to identify potential miRNA-181 targets with particular attention to proteins that are relevant to neuronal and synaptic plasticity. SIRT1 and the c-Fos transcription factor were identified as possible miRNA-181 targets. Both SIRT-1 and c-Fos are proteins involved in memory consolidation [95]. Protein levels of these potential targets evaluated in the ventral hippocampus of 3xTg-AD mice were significantly decreased. This result was also confirmed in the in vitro study, transfecting SH-SY5Y cells with miRNA-181. In this way, the downregulation of SIRT1 determined by the overexpression of miR-181 favors the development of Aβ. Similarly, reduced levels of c-Fos can contribute to cognitive impairment.

Instead, the miRNA-181c was studied by Zhou et al. [96]. For the study, SAMP8 mice were used and a significant decrease in miRNA-181c was observed in the hippocampus of SAMP8. Bioinformatics analysis revealed that miRNA-181c could be involved in the regulation of axon guidance, MAPK signaling, dorsal-ventral axis formation, and long-term depression. Furthermore, the results of a luciferase assay on HEK293 cells showed that miRNA-181c overexpression affects the 3′-UTR region of collapsin response mediator protein 2 (CRMP2). The CRMP2 protein is an intracellular protein expressed mainly in areas of high plasticities, such as the adult olfactory bulb, the cerebellum, and the hippocampus [97]. CRMP2 is also involved in the assembly of microtubules in neurons. Nevertheless, it seems that the hyperphosphorylation of CRMP2 protein is an early event in AD progression [98]. In order to further confirm whether miRNA-181c could regulate the translation of CRMP2, an in vitro study was also performed, transfecting miRNA-181c in HT-22 hippocampal neuronal cells. The results showed that the overexpression of miRNA-181c downregulates the expression of CRMP2 proteins. Consequently, the low expression of miRNA-181c observed in the hippocampus of SAMP8 mice could lead to an increase in the level of CRMP2 protein in AD mice, playing a potential role in the pathogenesis of AD.

Another miRNA involved in synaptic plasticity that plays a critical role in the memory deficits observed in patients with AD is miRNA-206. The aim of the study conducted by Tian et al. [99] was to determine the interaction between brain-derived neurotrophic factor (BDNF) and miRNA-206. BDNF is the most widely expressed neurotrophin in the central nervous system [100] and is involved in neurite growth, directional guidance, induction of long-term potentiation, and neurotransmitter release [101]. Several studies have shown that BDNF showed protective effect in AD both in in vivo [102] and in vitro experiments [103]. The results of the study showed that miRNA-206 was upregulated in hippocampal tissue, cerebrospinal fluid, and plasma of APP/PSEN1 transgenic mice. In order to understand how this miRNA is involved in BDNF alteration, BDNF levels have been assayed in mice. The results showed that the BDNF level in hippocampal tissue in APP/PSEN1 mice was decreased. In addition, researchers have shown that BDNF plays an important role in neuronal vulnerability and neuronal death. Primary neurons of the hippocampus from wild type and APP/PSEN1 mice were treated with extracellular Aβ_1–42_ or staurosporine or glutamate. The results induced more severe cell death in APP/PSEN1 neurons compared to wild type neurons. Subsequently, the administration of exogenous BDNF decreased the vulnerability in the primary neurons subjected to different types of insults, confirming its protective role of BDNF. Therefore, an alteration of miRNA-206 contributes to the pathology of AD through the downregulation of BDNF.

Macroautophagy is another process that can be regulated by miRNAs. It is a mechanism responsible for delivering cellular materials and organelles to lysosomes for degradation. In neurons, constitutive macroautophagy is involved in the maintenance of cellular homeostasis essential for cell survival [104]. On the contrary, induction of autophagy initiates a form of programmed cell death in some cellular populations [105], determining the loss of neural homeostasis that was responsible for AD. The purpose of the study conducted by Zhang et al. [106] is to investigate the role of miRNA-214-3p in the regulation of autophagy in SAD. In cerebrospinal fluid from patients with SAD was observed downregulation of miRNA-214-3p. In the in vivo experiment, the researchers showed the downregulation of miRNA-214-3p also in hippocampal neurons of SAMP8 mice. The in vitro study, transfecting the primary hippocampal neurons of SAMP8 with miRNA-214-3p, miRNA-214-3p inhibitor, or scrambled RNA as a negative control, showed that miRNA-214-3p overexpression in primary neurons from SAMP8 mice inhibited autophagy. Inhibition of autophagy was demonstrated by reduced levels of LC3βII and Beclin1, reduced number of autophagosome vesicles, and decrease of caspase-mediated apoptosis. In contrast, miRNA-214-3p inhibitors promoted autophagy and apoptosis in SAMP8 mice neurons. Finally, in order to evaluate the role of miRNA-214-3p in the regulation of autophagy and apoptosis, miRNA-214-3p or scrambled RNA (negative control, NC) were injected into SAMP8 mice. The results showed that the treatment with miRNA-214-3p had attenuated neuronal apoptosis and improved behavioral performance. These data suggest that the overexpression of miRNA-214-3p suppresses macroautophagy.

The study conducted by Li et al. [107] aimed to understand the role of miRNA-574 in early AD in 5-month APP/PSEN1 mice. The results showed a significant synaptic loss and high levels of Aβ_1–42_, with consequent cognitive deficits. The study also wanted to find out if there was a miRNA in the hippocampus linked to synapse-associated proteins in the early phase of AD development. The microarray miRNA assay was performed in the hippocampus tissue of wild type and APP/PSEN1 mice. The results showed that miRNA-382, miRNA-711, and miRNA-574 were upregulated in APP/PSEN1 mice, while the miRNA-335 was downregulated. Meanwhile, a bioinformatics analysis had predicted that, in particular, miRNA-574-5p could have a direct connection with neuritin (NRN1), and, therefore, in the function of synaptic plasticity. NRN1 is a neurotrophic factor involved in synaptic plasticity and neuritogenesis, which can play important roles in regeneration and repair following compromised nervous systems [108,109]. In order to observe the connection between synaptic loss and miRNA-574, the primary hippocampal neuron from the APP/PSEN1 transgenic mice was treated with the peptide fragment Aβ_25–35_. The results revealed a high expression of miRNA-574-5p demonstrating that the Aβ_25–35_ peptide could overregulate miRNA-574 expression. Successively, in order to test if miRNA-574-5p could influence the expression NRN1, HT22 hippocampal neuronal cells were transfected with miRNA-574-5p or miRNA-574-5p inhibitors. The results of the in vitro studies showed that miRNA-574 overexpression reduced the expression levels of the NRN1 protein. Conversely, NRN1 protein expression levels were significantly increased by transfecting cells with miRNA-574 inhibitors. All these data confirm the hypothesis that miRNA-574 targeting NRN1 may be directly responsible for the loss of synapses in the hippocampus of 5-month APP/PSEN1 mice.

Another miRNA that can contribute to the initiation and progression of AD is miRNA-222. In particular, the study conducted by Wang et al. [110] aimed at clarifying the interactions existing between miRNA-222 and P27^KIP1^ in AD. P27^KIP1^ is also called cyclin-dependent kinase 1B inhibitor and is responsible for blocking the cell cycle [111]. In addition, it appears that the overexpression of P27^KIP1^ is correlated with the pathogenesis of AD [112]. The in vivo study conducted on APP/PSEN1 mice showed that miRNA-222 levels were downregulated compared to control. In contrast, P27^KIP1^ protein levels were increased. In order to further determine whether miRNA-222 can directly regulate P27^KIP1^, the researchers also analyzed changes in the level of P27^KIP1^ in SH-SY5Y cells transfected with miRNA-222. Even in vitro results also confirmed an inverse correlation between the expression levels of P27^KIP1^ and miRNA-222. These results show how the downregulated expression of miRNA-222 influences cell cycle dysregulation in AD, by targeting P27^KIP1^.

miRNA-128 is another miRNA involved in the development and progression of AD. The downregulation of miRNA-128 seems to facilitate the degradation of Aβ_1–42_, suggesting the link between miRNA-128 and AD progression [113]. The study conducted by Liu et al. [114] aimed to study the roles and molecular mechanisms of miRNA-128 in the development and progression of AD. In the present study, it was shown that in the cerebral cortex of 3xTg-AD mice, the levels of miRNA-128 and Aβ was significantly increased compared to wild type mice. On the contrary, the proliferator-activated gamma receptor (PPARγ) level was downregulated. PPARγ plays crucial roles in multiple biological processes such as metabolism, morphogenesis, and inflammation and appears to be multiplied in the development of some neurodegenerative disorders including AD [115]. Furthermore, a large number of trials have shown that PPARγ agonists can improve the pathological condition of AD as they induce anti-inflammatory and anti-amyloidogenic effects [116,117]. In parallel, the researchers conducted an in vitro study, transfecting Neuro2A cells with miRNA-128 or miRNA-128 inhibitor. Even the results in vitro showed that miRNA-128 overexpression led to a marked reduction in the level of PPARγ protection in cells. In contrast, the PPARγ protein level was significantly increased in cells in which miRNA-128 was inhibited. Subsequently, 3xTg-AD mice with miRNA-128 knockout were used to further explore the roles and molecular mechanisms of miRNA-128 in AD development. The miRNA-128 level was notably reduced in the cerebral cortex of 3 × Tg-AD mice with a miRNA-128 knockout as compared to that in the cerebral cortex of 3 × Tg-AD mice. These mice with miRNA-128 showed improvement in cognitive abilities and reduced anxiety compared to 3 × Tg-AD mice corresponding to age. Furthermore, the formation of amyloid plaques and the generation of Aβ peptides were inhibited by inactivation of the APP amyloidogenic processing pathway. Therefore, miRNA-128 could exert its pathogenic activity by focusing on PPARγ; on the contrary, the inhibition of PPARγ could represent a possible therapeutic strategy.

The preclinical studies regarding the role of miRNAs in AD are summarized in Table 1.

## Figures and Tables

**Table 1:** A summary of microRNAs (miRNAs) linked to Alzheimer’s disease (AD).

miRNAs	Target mRNA	Models	Experimental Outcomes	Field of Application	Ref.
miRNA-101a-3p	APP	APP/ PSEN1 AD mice and SH-SY5Y cells	The overexpression of osthole-induced miRNA inhibits APP mRNA-101a-3p and reduces APP protein levels.	Therapeutic target	[40]
miRNA-200b/c	APP	Tg2576 mice; PMNCs and SH-SY5Y cells	miRNA-200b/c reduces Aβ secretion and Aβ-induced cognitive impairment. In addition, the transient transfection of neurons with miRNA-200b/c, decreased the secretion of Aβ in the conditioned medium.	Therapeutic targets	[43]
miRNA-200b; miRNA135a	APP; BACE1	APP/PSEN1 mice; primary neurons of the hippocampus mice, SH-SY5Y and HEK293 cells	miRNA-200b and miRNA-135a downregulated in hippocampi from APP/PS1 transgenic mice and repressed respectively the expression and activity of APP and BACE1.	Diagnostic markers	[44]
miRNA200a-3p	SIRT1	APP/PSEN1 AD mice and PC12 cells	Increased level of the miRNA-200a-3p and decreased level of SIRT1 in the hippocampus of APP/PS1 mice were observed. Downregulation of miRNA-200a-3p protected PC12 cells from Aβ_25–35_-induced neurotoxicity and inhibited the cell apoptosis. Moreover, SIRT1 was a target gene of miRNA-200a-3p and exerted a neuroprotective effect against Aβ_25–35_-induced toxicity in PC12 cell.	Therapeutic target	[45]
miRNA-29c	BACE1	SAMP8 and SAMR1 mice, primary hippocampal neurons of SAMR1 mice and peripheral blood of patients with AD	A decrease in miRNA-29c expression levels and a significant increase in BACE1 expression in peripheral blood samples from AD patients were recorded. miRNA-29c regulates BACE1 expression at the transcriptional level by directly targeting its 3’UTR. In SAMP8 mice miRNA-29c promoted a decrease in the production of Aβ by targeting BACE1.	Therapeutic target	[49]
miRNA-29c	NAV3	APP/PSEN1 AD mice and Neuro2A cells	miRNA-29c was significantly increased in the hippocampus of APP/PS1 mice, while it decreased in the frontal cortex. The differential expression of miRNA-29c in the hippocampus and frontal cortex of the APP/PS1 mouse brain was also accompanied by the inverse expression of the NAV3. In the in vitro study miRNA-29c directly mediated downregulation of NAV3 protein expression. miRNA-29c may influence neurodegenerative processes by targeting NAV3.	Therapeutic target	[50]
miRNA-137	CACNA1C	APP/PSEN1 AD mice and SH-SY5Y cells	The level of miRNA-137 decreased, while the level of CACNA1C increased in the hippocampus and cerebral cortex of AD mice. In SH-SY5Y cells inhibition of miRNA-137 also caused an increase in Ab1–42-induced hyperphosphorylation of TAU in SH-SY5Y cells.	Therapeutic target or diagnostic marker	[52]
miRNA-10a-5p, miRNA-142a-5p, miRNA-146a-5p, miRNA-155-5p, miRNA-211- 5p and miRNA-455-5p	-	APPtg and TAUtg mice	Upregulation between APPtg and TAUtg mice of miRNA-10a-5p, miRNA-142a-5p, miRNA-146a-5p, miRNA-155-5p, miRNA-211-5p, miRNA-455-5p; and upregulation of four of these (miRNA-142a-5p, miRNA-146a-5p, miRNA-155-5p, and miRNA-455-5p) also in AD patients.	Diagnostic markers	[57]
miRNA-330	VAV1	C57BL/6J AD mice and primary neuronal cells	Overexpression of miRNA-330 decreases expression of VAV1 via the MAPK pathway, reducing Aβ production, alleviates oxidative stress and mitochondrial dysfunction.	Therapeutic target	[58]
miRNA-34a	GRM7	3xTg-AD mice	Upregulated expression of miRNA-34a could be attributed to anxiety-like behaviors in 3xTg-Ad mice and linked to the downregulation of anxiety-related target gene GRM7.	Diagnostic markers	[68]
miRNA-34a	-	APP/PSEN1 and miRNA-34a KO/APP/PS1 mice	The level of miRNA-34a was increased, according to the increase in amyloid β (Aβ) in APP/PS1 mice; instead, in miRNA-34a knockout mice, a significantly reduced behavioral dysfunction was observed, mainly by inhibiting the γ-secretase activity.	Diagnostic markers	[63]
miRNA-34a	AMPA and NMDA	APP/PSEN1 and miRNA-34a KO/APP/PS1 mice	miRNA-34a deficiency promotes cognitive function by increasing synaptic plasticity via AMPA and NMDA receptors.	Diagnostic markers	[64]
miRNA-302/367	-	C57BL/6J AD mice	The miRNA-302/367 overexpression allows activated astrocytes to be converted into neurons by restoring some aspects of learning and memory deficits in an animal model of AD.	Therapeutic target	[71]
miRNA-125b, miRNA-181c, miRNA-9 and miRNA-191-5p	-	APP/PSEN1 AD mice and SH-SY5Y	Serum miRNA-125b, miRNA-191-5p, miRNA-9 were significantly downregulated and miRNA-28-3p was upregulated in APP/PS1 transgenic mice. Instead, the level of serum miRNA-125b, miRNA-9, and miRNA191-5p were upregulated in EGCG-treated APP/PS1.	Diagnostic markers	[74]
miRNA-139	CB2	SAMP8 and SAMR1 mice	miRNA139 expression was significantly higher in SAMP8 mice, compromising hippocampal-dependent learning and memory formation. In contrast, the downregulation of miRNA-139 in mice improved learning and memory in mice. Furthermore, miRNA-139, by inhibiting CB2 expression, decreases responses to pro-inflammatory stimuli and acts as a regulatory factor in the pathogenesis of AD.	Therapeutic target	[78]
miRNA-188-5p	-	5xFAD mice AD model, primary hippocampal neuron cells and human AD brains	The expression of miRNA-188-5p was downregulated in brain tissues from patients with AD and 5XFAD mice. Treatment with oligomeric Aβ_1–42_ decreased miRNA-188-5p expression in primary cultures of hippocampal neurons. On the contrary, miRNA-188-5p overexpression could alleviate the decrease in dendritic spine density in primary hippocampal neurons exposed to Aβ. Therefore, replenishment of mi-R188-5p restores the synaptic and cognitive deficits.	Diagnostic marker and therapeutic target	[82]
miRNA-132	ITPKB	APP/PSEN1 AD mice	MiRNA-132 loss aggravates amyloid and TAU pathology in AD brain via ITPKB upregulation in AD mice model. This lead to increased ERK1/2 and BACE1 activity and elevated TAU phosphorylation. Downregulation of miRNA132 and upregulation of ITPKB was confirmed in human AD patients.	Diagnostic marker and therapeutic target	[85]
miRNA-132/212	TAU	3xTG-AD mice and mouse Neuro2a cells	Deletion of miRNA-132/212 caused abnormal TAU metabolism, accentuate TAU hyperphosphorylation and TAU aggregation. TAU is a direct target of miRNA-132. On the contrary, the treatment of 3xTg-AD mice with miRNA-132 mimics improved a long-term memory deficit and was determinate a significant reduction of phosphorylated TAU.	Therapeutic target	[88]
miRNA-132/212	SIRT1	3xTG-AD mice and mouse Neuro2a cells	Genetic deletion of miRNA-132/212 promotes Aβ production and amyloid plaque formation. SIRT1, MAPK1 / ERK2, and TAU were interesting miRNA-132 targets identified that were found to be upregulated in 3xTg-AD mice compared to controls. In contrast, all these genes were downregulated in Neuro2a132 cells. In addition, the modulation of miRNA-132 or SIRT1 can directly regulate Aβ production in cells.	Therapeutic target	[91]
miRNA-181	SIRT1 and c-Fos	3xTG-AD mice and SH-SY5Y cells	Compared to twelve-month wild type mice in the hippocampus of age-matched 3xTg-AD mice with plaques and tangles was found a significant upregulation of miRNA-181. Analysis of predicted targets of miRNA-181 identified c-Fos and SIRT-1. Both c-Fos and SIRT-1 levels were significantly decreased in the ventral hippocampus of twelve-month old 3xTg-AD mice. In addition, overexpression of miRNA-181 in SH-SY5Y cells significantly decreased c-Fos and SIRT-1.	Diagnostic marker and therapeutic target	[94]
miRNA-181c	CRMP-2	SAMP8 and SAMR1 mice; HT-22 and HEK293A cells	A significant decrease in miRNA-181c in the hippocampus of SAMP8 was recorded. miRNA-181c overexpression affects the 3’-UTR region of CRMP2. In HT-22 hippocampal neuronal cells, the overexpression of miRNA-181c downregulates the abundance of CRMP2 proteins at the post-transcriptional level.	Diagnostic marker	[96]
miRNA-206	BDNF	APP/PSEN1 mice and primary neuron cells from embryonic APP/PSEN1 mice hippocampus	miRNA-206 was upregulated, this overexpression resulted in a downregulated expression of BDNF that protects against cell death.	Diagnostic marker	[99]
miRNA-214-3p	ATG12	SAMR1 and SAMP8 mice; primary neuron cells from embryonic SAMP8 mice hippocampus and SH-SY5Y cells	Downregulated miRNA-214-3p was observed in hippocampal neurons of SAMP8 mice and also in cerebrospinal fluid from patients with SAD. miRNA-214-3p overexpression in primary neurons from SAMP8 mice inhibited autophagy. In contrast, antagomiRNA-214-3p promoted macroautophagy and apoptosis in SAMP8 mice neurons. miRNA-214-3p by directly targeting ATG12 inhibits the macroautophagy. In addition, the injection of miRNA-214-3p into the hippocampal improved the cognitive capacity of SAMP8 mice.	Therapeutic target	[106]
miRNA-146-a	ROCK1	5xFAD mice and SH-SY5Y cells	High levels of miRNANA-146a in neurons negatively regulate the translation of the ROCK1 protein. Reduction of neuronal protein ROCK1 leads to a reduction in the neuronal phosphorylation of PTEN resulting in the impaired dephosphorylation of neuronal TAU.	Diagnostic marker and therapeutic target	[92]
miRNA-574	NRN1	APP/PSEN1 mice; primary hippocampal neuron from the wild type and APP/PS1 transgenic mice and HT22 hippocampal neuronal cells	miRNA-574 was significantly increased in the hippocampus of 5-month APP/PS1 mice, showing synaptic loss and cognitive impairment. Bioinformatic analysis predicted that mRNA-574 targets the mRNA of Nrn1. In fact, in the in vitro study, miRNA-574 overexpression reduced NRN1 expression levels. In contrast, miRNA-574 suppression by the miRNA-574 inhibitor resulted in elevated levels of NRN1 expression.	Diagnostic marker and therapeutic target	[107]
miRNA-222	P27^KIP1^	APP/PSEN1 mice, SH-SY5Y and HEK-293T	Downregulation expression of miRNA-222 influences cell cycle dysregulation in AD, by targeting P27^KIP1^	-	[110]
miRNA-128	PPARγ	3xTG-AD mice and Neuro2A cells	The expression of miRNA-128 was upregulated, on the contrary, the expression of PPARγ was downregulated in the cerebral cortex of AD mice. Furthermore, PPARγ was a target of miRNA-128. In addition, the upregulation of miRNA-128 or the upregulation of PPARγ inhibited type AD performance, the formation of amyloid plaques, the generation of Aβ, the amyloidogenic processing of APP, and the inflammatory responses in AD mice. Instead, the inhibition of PPARγ improves the effects caused by miRNA-128.	Diagnostic marker and therapeutic target	[114]

## Abbreviations

AD	Alzheimer’s disease
Aβ	Amyloid-beta
NFTs	Neurofibrillary tangles
FAD	Familial Alzheimer’s disease
SAD	Sporadic Alzheimer’s disease
APP	Amyloid precursor protein
BACE1	β-site amyloid precursor protein cleaving enzyme
PSEN1	Presenilin 1
PSEN2	Presenilin 2
APOE	Apolipoprotein E
LRP1	Protein 1 associated with the LDL receptor
UTR	Untranslated region
mRNAs	Messenger RNAs
Pri-miRNAs	Primary miRNAs
Pre-miRNAs	Precursor miRNAs
RISC	RNA-induced silencing complex
SH-SY5Y	Human neuroblastoma
PMNCs	Primary murine neuronal cells
qRT-PCR	Quantitative reverse transcription PCR
HEK293	Human embryonic kidney 293
SIRT1	Silent information regulator transcript-1
Ca^2+^	Calcium
VAV1	Guanine nucleotide exchange factor 1
MAPK	Mitogen-activated protein kinase
3xTg-AD	Triple transgenic mice model of AD
GRM7	Metabotropic glutamate receptor 7
miRNA-34a KO/APP/PS1	miRNA-34a knockouts APP/PSEN1 mice
AMPA	α-amino-3-hydroxy-5-methyl-4-isoxazolepropionic acid
NMDA	*N*-methyl-d-aspartate
STZ	Streptozotocin
VPA	Valproate
EGCG	Epigallocatechin gallate
SAMP8	Senescence-accelerated mouse prone 8
SAMR1	Senescence-accelerated mouse-resistant 1
CB2	Cannabinoid receptor type 2
ITPKB	Inositol 1,4,5-trisphosphate 3-kinase B
Neuro2a	Neuroblastoma 2a
Neuro2a132	Neuro2a cells treated with miRNA-132 mimics
Crmp2	Collapsin response mediator protein 2
BDNF	Brain-derived neurotrophic factor
ROCK1	Coiled-coil containing protein kinase 1
PTEN	Phosphatase and tensin homolog
NAV3	Navigator neuron 3
Nrn1	Neuritin
PPARγ	Proliferator-activated receptor gamma
